# Risk factors for delayed gastric emptying in pancreaticoduodenectomy

**DOI:** 10.1038/s41598-022-26814-7

**Published:** 2022-12-23

**Authors:** Shih-Hao Mao, Bor-Shiuan Shyr, Shih-Chin Chen, Shin-E Wang, Yi-Ming Shyr, Bor-Uei Shyr

**Affiliations:** 1 Division of General Surgery, Department of Surgery, Taipei Veterans General Hospital, 201 Section 2 Shipai Road, Taipei, 112 Taiwan, ROC; 2 National Yang Ming Chiao Tung University, Taipei, Taiwan, ROC

**Keywords:** Cancer, Gastroenterology, Medical research

## Abstract

The study of robotic pancreaticouodenectomy (RPD) focusing on delayed gastric emptying (DGE) is seldom reported. This study explored the incidence of DGE in RPD with extracorporeal hand-sewn gastrojejunostomy involving downward positioning of the stomach. Patients with periampullary lesions undergoing RPD or open pancreaticouodenectomy (OPD) were included for comparison. A variety of clinical factors were evaluated for the risk of developing DGE. There were 409 (68.2%) RPD and 191 (31.8%) OPD in this study. DGE occurred in 7.7% of patients after pancreaticoduodenectomy, with 4.4% in RPD and 14.7% in OPD, *p* < 0.001. Nausea/vomiting (12.6% vs. 6.3%) and jaundice (9.9% vs. 5.2%) were significant preoperative risk factors for DGE, while malignancy (8.7% vs. 2.2%) and lymph node involvement (9.8% vs. 5.6%) were significant pathological risk factors. Intraoperative blood loss > 200 c.c. was the other factor related to DGE (11.2% vs. 4.4% in those with blood loss ≤ 200 c.c.). None of the postoperative complications was significantly associated with DGE. Hospital stay was significantly longer in the group with DGE (median, 37 vs. 20 days in the group without DGE). After multivariate analysis by binary logistic regression, compared with OPD, RPD was the only independent factor associated with a lower incidence of DGE. RPD with extracorporeal hand-sewn antecolic, antiperistaltic, and inframesocolic gastrojejunostomy via a small umbilical wound involving careful downward positioning of the stomach was associated with a low incidence of DGE and presented as the most powerful independent predictor of this condition.

## Introduction

Pancreaticoduodenectomy is a challenging abdominal surgery with a high morbidity rate of 40% to 57%, although the mortality rate after this procedure has recently improved to < 5% in high-volume centers^1–4^. Postoperative pancreatic fistula (POPF) is the most dreaded complication of pancreaticoduodenectomy, whereas delayed gastric emptying (DGE) has been identified as one of the most common causes of morbidity following conventional open pancreaticoduodenectomy (OPD)^1,3–7^. DGE is characterized by a failure to progress with an appropriate diet, and symptoms of nausea and vomiting resulting from postoperative gastroparesis without apparent anatomic strictures or obstructions^8^. Although not imminently life-threatening, DGE is a bothersome complication, which can lead to nutritional difficulty, prolonged length of hospital stay, decreased quality of life, delayed adjuvant chemotherapy, and increased healthcare costs^9,10^.

However, the incidence of DGE after pancreaticoduodenectomy ranges widely from 6 to 57%, because of the heterogeneity in the surgical procedures, number of surgeons involved, and varying definitions of DGE^1–5,7,8^. There was wide variability in the definition of DGE until consensus criteria were established for DGE in 2007 by the International Study Group of Pancreatic Surgery (ISGPS)^11^. The association between the operative technique and DGE, specifically pylorus-preservation pancreaticoduodenectomy (PPPD) versus classic pancreaticoduodenectomy, and antecolic versus retrocolic gastrojejunostomy, has been studied in a number of retrospective studies with conflicting reports^1^. With the introduction of the da Vinci Surgical System (Intuitive Surgical, Inc., Sunnyvale, CA, USA), minimally invasive surgery has dramatically changed the method of several surgical procedures^7,12,13^. Our pancreas team has been performing robotic pancreaticoduodenectomy (RPD) since 2014 and has established its feasibility, safety, and justification as equal to or superior to that of OPD^14–16^. However, studies of RPD focusing on DGE is seldom reported^7^.

This study was conducted to identify pre-, intra- and post-operative factors associated with the development of DGE after RPD. OPD was performed by one surgical team using the same surgical techniques, with the aim of predicting those patients at greater risk, in order to develop preventive strategies for DGE. Moreover, this study aimed to clarify the incidence of DGE in RPD with extracorporeal hand-sewn gastrojejunostomy involving careful downward positioning of the stomach.

## Materials and methods

Data of patients with periampullary lesions undergoing RPD or OPD between July 2014 and August 2021 were identified from a prospectively collected database. This study was approved by the Institutional Review Board of Taipei Veterans General Hospital, (IRB-TPEVGH No.: 2021-11-006AC), and carried out in accordance with the IRB guidelines and regulations. The requirement for informed consent was waived in this retrospective cohort study with data anonymity by the Institutional Review Board of Taipei Veterans General Hospital. Patient selection for RPD was determined by patient preferences, after detailed counselling about the innovative nature of RPD as well as its advantages and disadvantages and the availability of a robotic machine. Patients with previous upper abdominal surgery which could be associated with severe adhesion and long vascular encasement more than 2 cm were not considered for RPD. Patient demographic and clinical variables were assessed, which included sex, age, body mass index (BMI), diabetes mellitus (DM), American Society of Anesthesiologists (ASA) physical status classification, clinical presentations, and diagnosis. Additionally, pathological variables, such as malignancy, tumor size, lymph node status, perineural invasion, lymphovascular invasion, and stage were included for evaluation. Intraoperative variables were surgical approach (RPD vs. OPD), PPPD, operation time, blood loss, vascular resection, and tumor radicality. Postoperative variables for evaluation included surgical mortality and a variety of complications after pancreaticoduodenectomy.

### Study endpoints

The primary study endpoint was to clarify the incidence of DGE in RPD with extracorporeal hand-sewn gastrojejunostomy involving careful downward positioning of the stomach, while the secondary study endpoint was to identify the risk factors associated with DGE after RPD and OPD.

### Surgical technique

RPD and OPD were performed with the same surgical technique by the same team led by Shyr YM. All the RPDs were carried out with the assistance of Si or da Vinci Xi Robotic Surgical System (Intuitive Surgical, Inc., Sunnyvale, CA, USA). There were some differences in the technique used between the OPD and RPD groups. In the RPD group, the Harmonic® scalpel, an energy device, was used for the small vessel division, and Hem-o-lok® systems (Teleflex Inc., Chelmsford, MA, USA) were selectively for the large vessels. Most of the vascular pedicles were cauterized or selectively ligated in the OPD.

**Figure 1:**
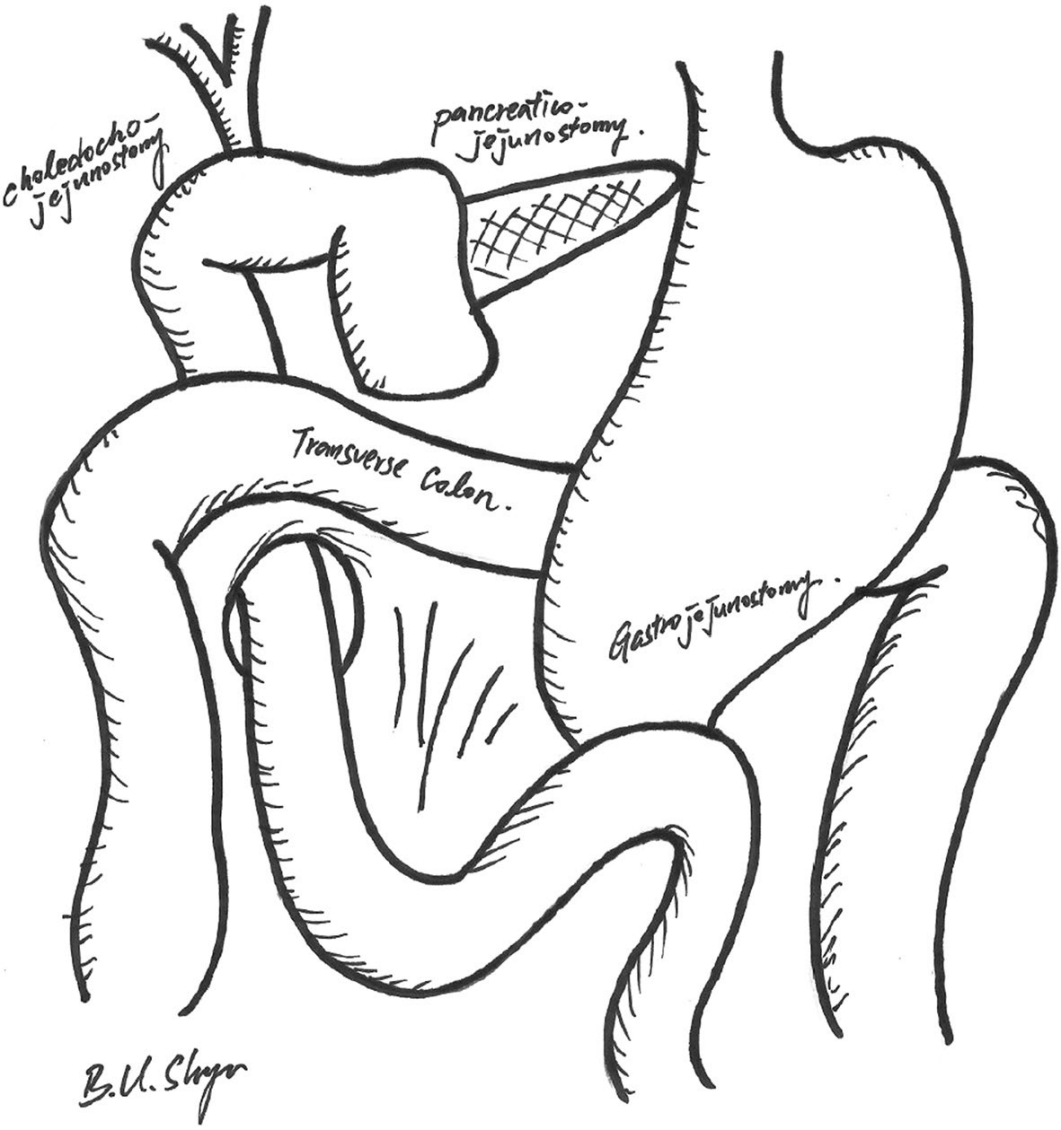
Sketch illustration of surgical technique in RPD with antecolic, antiperistaltic, and inframesocolic gastrojejunostomy. The influence of inflammation created after pancreaticoduodenal resection or related to the pancreatic fistula on the gastrojejunal anastomosis could be avoided or minimized.

**Figure 2:**
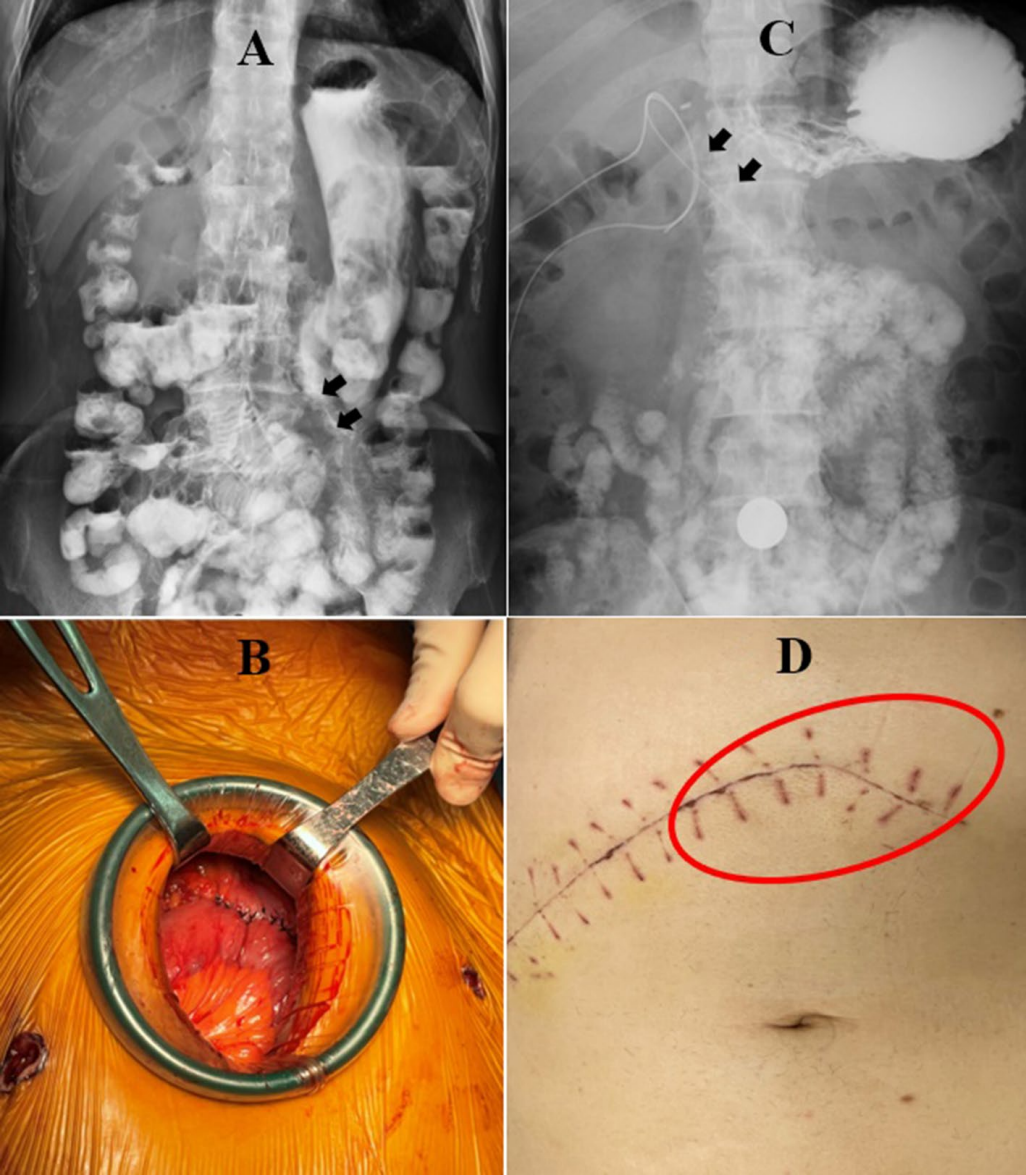
(**A**) The relatively vertical and straight stomach in position and the gastrojejunostomy (indicated by 2 black arrows) near the umbilicus region. (**B**) The last anastomosis in RPD hand-sewn gastrojejunostomy performed using the extracorporeal approach, involving careful downward positioning of the stomach. The final position of stomach and gastrojejunstomy is antecolic, antiperistaltic, and inframesocolic. (**C**) The relatively horizontal stomach in position and the gastrojejunostomy (indicated by 2 black arrows) near the pancreaticojejunostomy (indicated by 2 opacified drains) and above the transverse colon (colon gas inside) after OPD. (**D**) The large abdominal incision wound after OPD. The jejunal limb is pulled upward for gastrojejunal anastomosis, and the final position of the stomach (indicated by a red ovoid circle) and gastrojejunostomy is antecolic, antiperistaltic, and supramesocolic, just beneath the large abdominal incisional wound.

After a standard pancreaticoduodenectomy, reconstruction was completed involving an end-to-side pancreaticojejunostomy, end-to-side hepaticojejunostomy, and end-to-side gastrojejunostomy on the same jejunal limb. The pancreatic reconstruction was completed with a modified Blumgart pancreaticojejunostomy described previously in detail^15,17,18^. In the RPD group, all the resected specimen were extracted through a 4–6 cm umbilical wound. To facilitate anastomosis and save time, the small umbilical wound was also used for the extracorporeal hand-sewn gastrojejunostomy involving a careful downward positioning of the stomach. The final position of gastrojejunstomy was antecolic, antiperistaltic, and inframesocolic near the umbilicus region (Fig. 1). Therefore, the stomach was in a relatively vertical position after the RPD (Fig. 2A, B). In the OPD group, the jejunal limb was pulled upward for the gastrojejunostomy, and the final position was antecolic, antiperistaltic, and supramesocolic. Thus, after OPD, the stomach was relatively horizontal in position, and just beneath the large abdominal incisional wound (Fig. 2C, D).

The right gastric artery was routinely divided in our practice. PPPD was attempted initially whenever possible; otherwise, a limited antrectomy was done for those with ischemic pylorus after dividing the right gastric artery. After surgery, intravenous proton pump inhibitors were routinely administered to all the patients. Upon the resumption of food intake, medications were administered orally. Prophylactic octreotide or prokinetic drugs were not used after surgery. The nasogastric tube (NGT) that was routinely placed before surgery, was removed if the volume of the NGT drainage was ≤ 300 c.c. and there were no gastrointestinal disturbances. Oral intake with a clear liquid diet was started, usually before postoperative day (POD) 3. Abdominal X-ray after oral contrast intake was conducted for those with suspicious DGE.

### Definitions of surgical complications

In this study, DGE was referred to as a clinically significant grade B or C, based on the criteria proposed by the ISGPS^11^. DGE was subdivided into ISGPS grade A, B, and C in order of increasing severity. Grade A was defined as requiring an NGT within POD 4–7, reinsertion of the NGT after removal on POD 3, or inability to tolerate a solid diet by POD 7. Grade B was defined as requiring NGT from POD 8–14, reinsertion of the NGT after POD 7, or inability to tolerate a solid diet by POD 14. Lastly, grade C was defined as the inability to discontinue the NGT, reinsertion of the NGT after POD 14, or inability to tolerate a solid diet by POD 21.

POPF was referred to in terms of a clinically relevant grade B or C pancreatic leakage, based on the 2016 new grading system by the International Study Group for Pancreatic Fistula^19^. Postpancreatectomy hemorrhage (PPH) and chyle leak were classified using the standardized criteria proposed by the ISGPS^20,21^. Resection radicality was stratified into three categories based on the resection margin status: R0, cancer-free margin without gross and microscopic evidence of cancer cells at the resection margin, under a definition of margin > 0 mm, instead of 1 mm, defined by the National Comprehensive Cancer Network; R1, a resection with microscopically positive cancer cells at the resection margin, but grossly negative; and R2, a resection with grossly positive cancer cells at the resection margin. Therefore, R0 was curative PD, while both R1 and R2 were palliative PD in this study. Surgical mortality was defined as death within 90 days after surgery, including the same period of admission and hospital readmission after the operation.

### Statistical analysis

Statistical analysis was carried out by Statistical Product and Service Solutions version 21.0 software (SPSS Inc, IBM, Armonk, NY, USA). The mean continuous variables distributed normally were compared between groups using the two-tailed Student’s *t* test. The Wilcoxon rank-sum test was used for continuous variables without normal distribution. All continuous data were presented as median (range) and mean ± standard deviation. Nonparametric statistical tests were used if the variables did not follow a normal distribution. Categorical variables were presented as the number (percentage) and compared using Pearson’s χ^2^ test or Fisher’s exact test contingency tables. Variables that were considered significant (*p* < 0.05) in the univariate analysis were entered into the multivariate analysis to perform binary logistic regression. Confidence intervals were set at 95% and a *p* value of < 0.05 was considered statistically significant.

## Results

A total of 600 patients with periampullary lesions undergoing pancreaticoduodenectomy were enrolled in the study, including 234 (39.0%) with pancreatic head adenocarcinoma, 129 (21.5%) with ampullary adenocarcinoma, 36 (6.0%) with distal common bile duct adenocarcinoma, 28 (4.7%) with duodenal adenocarcinoma, 80 (13.3%) with other malignancies, 70 (11.7%) with other benign lesions, and 23 (3.8%) with chronic pancreatitis. Forty-six (7.7%) patients were associated with DGE after pancreaticoduodenectomy. DGE occurred in 46 (7.7%) patients after pancreaticoduodenectomy. Preoperative and demographic factors are listed in Table 1. Patients presenting with nausea/vomiting tended to have DGE, 12.6% versus 6.3%, *p* = 0.019. Patients with preoperative jaundice were associated with a higher rate of DGE than those without, 9.9% versus 5.2%, respectively, *p* = 0.023. Sex, age, DM, BMI, ASA physical status classification, body weight loss, and type of periampullary lesions were not predictors of DGE. There were 70 cases classified to be other benign lesion, and none of these benign diseases was associated with DGE.

**Table 1:** Preoperative and demographics factors for patients with periampullary lesions undergoing pancreaticoduodenectomy.

	Total	DGE (+)	DGE (−)	*p* value
Patients, n (%)	600	46 (7.7%)	554 (92.3%)	
**Sex**				0.289
Female	283 (47.2%)	24 (8.5%)	259 (91.5%)	
Male	317 (52.8%)	22 (6.9%)	295 (93.1%)	
**Age, year old**				0.583
Median (range)	66 (19 – 97)	66 (43 – 89)	66 (19 – 97)	
Mean ± SD	65.1 ± 12.2	66.1 ± 10.5	65.1 ± 12.3	
**Nausea/vomiting**				0.019
Yes	127 (21.2%)	16 (12.6%)	111 (87.4%)	
No	473 (78.8%)	30 (6.3%)	443 (93.7%)	
**Diabetes mellitus**				0.144
Yes	162 (27.0%)	16 (9.9%)	146 (90.1%)	
No	438 (73.0%)	30 (6.8%)	408 (93.2%)	
**BMI**				0.455
Median (range)	23.2 (14.5–36.2)	22.7 (16.9–32.9)	23.3 (14.5–36.2)	
Mean ± SD	23.5 ± 3.5	23.1 ± 3.6	23.5 ± 3.5	
**ASA physical status classification**				0.307
< 2	417 (69.5%)	30 (7.2%)	387 (92.8%)	
> 3	183 (30.5%)	16 (8.7%)	167 (91.3%)	
**Jaundice**				0.023
Yes	314 (52.3%)	31 (9.9%)	283 (90.1%)	
No	286 (47.7%)	15 (5.2%)	271 (94.8%)	
**Body weight loss**				0.303
Yes	170 (28.3%)	15 (8.8%)	155 (91.2%)	
No	430 (71.7%)	31 (7.2%)	399 (92.8%)	
**Periampullary lesions**				0.189
Pancreatic head adenocarcinoma	234 (39.0%)	21 (9.0%)	213 (91.0%)	
Ampullary adenocarcinoma	129 (21.5%)	12 (9.3%)	117 (90.7%)	
Distal CBD adenocarcinoma	36 (6.0%)	1 (2.8%)	35 (97.2%)	
Duodenal adenocarcinoma	28 (4.7%)	2 (7.1%)	26 (92.9%)	
Other malignancy	80 (13.3%)	8 (10.0%)	72 (90.0%)	
Other benign lesion	70 (11.7%)	0	70 (100%)	
Chronic pancreatitis	23 (3.8%)	2 (8.7%)	21 (91.3%)	

*DGE* delayed gastric emptying, *SD* standard deviation, *BMI* body mass index, *ASA* American Society of Anesthesiologists, *CBD* common bile duct.

Histopathological factors are shown in Table 2. Malignancy was a significant factor related to DGE, which occurred in 8.7% patients, as compared with only 2.2% in patients with a benign lesion, *p* = 0.016. Patients with lymph node involvement had a higher rate (9.8%) of DGE than those without (5.6%), *p* = 0.035. Other histopathological factors were not significantly associated with DGE, including tumor size, lymph node yield, perineural invasion, lymphovascular invasion, and stage.

**Table 2:** Histopathological factors for DGE after pancreaticoduodenectomy.

	Total	DGE (+)	DGE (−)	*p* value
Patients, n (%)	600	46 (7.7%)	554 (92.3%)	
**Malignancy**				0.016
Yes	507 (84.5%)	44 (8.7%)	463 (91.3%)	
No	93 (15.5%)	2 (2.2%)	91 (97.8%)	
**Tumor size, cm**				0.223
Median (range)	3.0 (0.5–33.5)	3.0 (0.8–5.0)	3.0 (0.5–33.5)	
Mean ± SD	3.4 ± 2.3	3.0 ± 1.0	3.4 ± 2.6	
**Lymph node involvement**				0.035
Yes	295 (49.2%)	29 (9.8%)	266 (90.2%)	
No	305 (50.8%)	17 (5.6%)	288 (94.4%)	
**Lymph node yield**				0.924
Median (range)	16 (5–49)	15 (7–28)	16 (5–49)	
Mean ± SD	17 ± 6	17 ± 6	17 ± 6	
**Perineural invasion**				0.219
Yes	339 (56.5%)	29 (8.6%)	310 (91.4%)	
No	261 (43.5%)	17 (6.5%)	244 (93.5%)	
**Lymphovascular invasion**				0.107
Yes	306 (51.0%)	28 (9.2%)	278 (90.8%)	
No	294 (49.0%)	18 (6.1%)	276 (93.9%)	
**Stage**				1.000
I and II	365 (74.3%)	31 (8.5%)	334 (91.5%)	
III and IV	126 (25.7%)	11 (8.7%)	115 (91.3%)	

*DGE* delayed gastric emptying, *SD* standard deviation.

**Table 3:** Intraoperative factors for DGE after pancreaticoduodenectomy.

	Total	DGE ( +)	DGE (-)	*p* value
Patients, n	600	46 (7.7%)	554 (92.3%)	
**Surgical approach**				< 0.001
RPD	409 (68.2%)	18 (4.4%)	391 (95.6%)	
OPD	191 (31.8%)	28 (14.7%)	163 (85.3%)	
**PPPD**				0.251
Yes	304 (50.7%)	26 (8.6%)	278 (91.4%)	
No	296 (49.3%)	20 (6.8%)	276 (93.2%)	
**Operation time, hour**				0.671
Median (range)	7.5 (3.3–16.3)	8.0 (4.5–16.3)	7.5 (3.3–15.3)	
Mean ± SD	7.9 ± 2.0	8.0 ± 2.1	7.9 ± 2.0	
**Blood loss**				0.001
< 200 c.c	315 (52.5%)	14 (4.4%)	301 (95.6%)	
> 200 c.c	285 (47.5%)	32 (11.2%)	253 (88.8%)	
**Vascular resection**				0.416
Yes	80 (13.3%)	7 (8.8%)	73 (91.3%)	
No	520 (86.7%)	39 (7.5%)	481 (92.5%)	
**Radicality**				0.976
R0	556 (92.7%)	43 (7.7%)	513 (92.3%)	
R1	15 (2.5%)	1 (6.7%)	14 (93.3%)	
R2	29 (4.8%)	2 (6.9%)	27 (93.1%)	

*DGE* delayed gastric emptying, *RPD* robotic pancreaticoduodenectomy, *OPD* open pancreaticoduodenectomy, *PPPD* pylorus-preserving pancreaticoduodenectomy, *SD* standard deviation, *R0* curative resection without residual cancer, *R1* microscopic residual cancer, *R2* gross residual cancer.

Intraoperative factors related to the surgery are included in Table 3. RPD was associated with only 4.4% of DGE, which is much lower than 14.7% of OPD, *p* < 0.001. Intraoperative blood loss > 200 c.c. was the other factor related to DGE, occurring in 11.2%, as compared with 4.4% of those with blood loss ≤ 200 c.c., *p* = 0.001. PPPD was performed in 304 (50.7%) patients, and proportion of PPPD in RPD group was higher than that in OPD, 59.7% versus 31.4%, *p* < 0.001, but the PPPD was not significantly associated with DGE, *p* = 0.251. In RPD group, DGE occurred in 13 (5.3%) patients with PPPD and 5 (3.0%) without PPPD, *p* = 0.266. In OPD group, DGE occurred in 13 (21.7%) patients with PPPD and 15 (11.5%) without PPPD, *p* = 0.064. In the group with PPPD, DGE occurred in 13 (5.3%) RPD patients and 13 (21.7%) OPD patients, *p* < 0.001. In the group without PPPD, DGE occurred in 5 (3.0%) RPD patients and 15 (11.5%) OPD patients, *p* = 0.004. Other factors intraoperative factors such as operation time, vascular resection, and tumor radicality were not significant predictors of DGE.

Postoperative factors related to complications after pancreaticoduodenectomy are shown in Table 4. The overall surgical mortality rate was 1.8%. The morbidity rate was 57%, with 12.2% comprising POPF, 5.8% PPH, 23.3% chyle leakage, 1.5% bile leakage, and 6.0% wound infection. However, none of these postoperative complications was significantly associated with DGE. Hospital stay was significantly longer in the group with DGE than that without, 37 versus 20 days (median), *p* < 0.001.

**Table 4:** Postoperative factors for DGE after pancreaticoduodenectomy.

	Total	DGE ( +)	DGE (-)	*p* value
Patients, n	600	46 (7.7%)	554 (92.3%)	
**Surgical mortality**				0.587
Yes	11 (1.8%)	1 (9.1%)	10 (90.9%)	
No	589 (98.2%)	45 (7.6%)	544 (92.4%)	
**POPF**				1.000
Yes	73 (12.2%)	6 (7.8%)	68 (92.2%)	
No	527 (87.8%)	40 (6.8%)	486 (93.2%)	
**PPH**				0.505
Yes	34 (5.8%)	2 (5.9%)	32 (94.1%)	
No	565 (94.2%)	44 (7.8%)	521 (92.2%)	
**Chyle leakage**				0.207
Yes	140 (23.3%)	7 (5.0%)	133 (95.0%)	
No	460 (76.7%)	39 (8.5%)	421 (91.5%)	
**Bile leakage**				0.515
Yes	9 (1.5%)	1 (11.1%)	8 (88.9%)	
No	591 (98.5%)	45 (7.6%)	546 (92.4%)	
**Wound infection**				0.466
Yes	36 (6.0%)	2 (5.6%)	34 (92.2%)	
No	564 (94.0%)	44 (7.8%)	520 (92.2%)	
**Hospital stay, day**				< 0.001
Median (range)	21 (6–136)	37 (13–61)	20 (6–136)	
Mean ± SD	24 ± 15	36 ± 11	23 ± 14	

*DGE* delayed gastric emptying, *POPF* postoperative pancreatic fistula, *PPH* postpancreatectomy hemorrhage, *SD* standard deviation.

**Figure 3:**
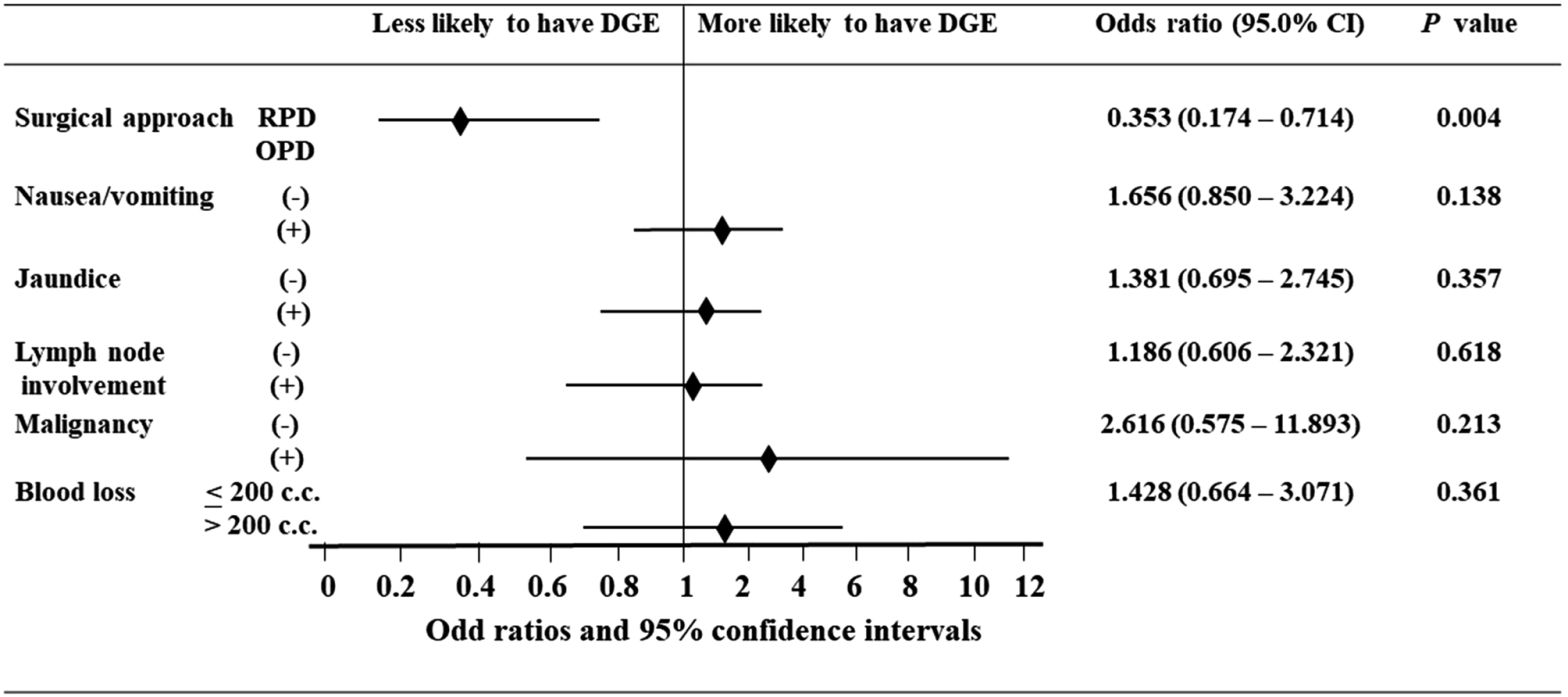
Forest plot, multivariate analysis by binary logistic regression for independent factors associated with delayed gastric emptying (DGE). CI: confidence interval, RPD: robotic pancreaticoduodenectomy, OPD: open pancreaticoduodenectomy.

**Figure 4:**
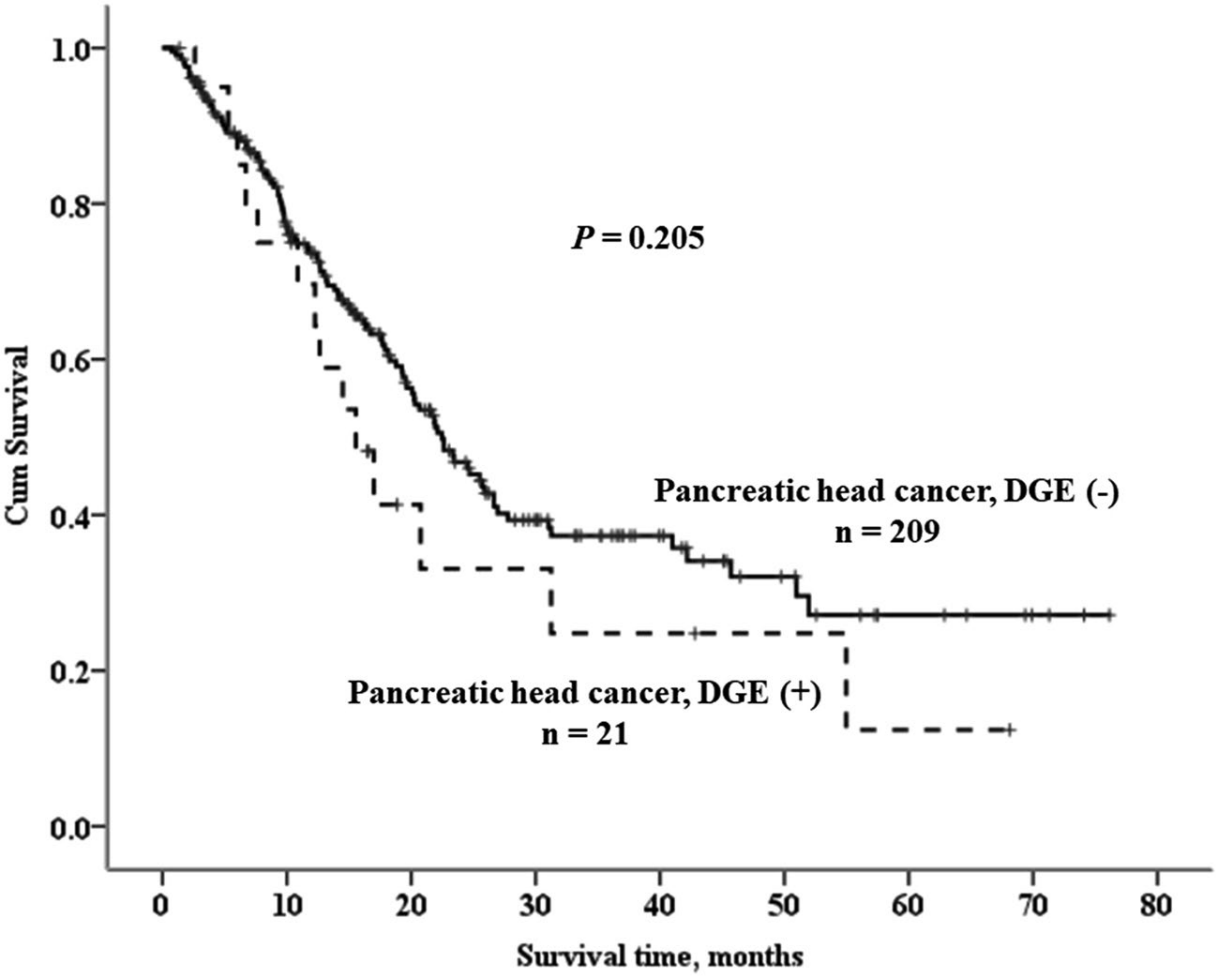
Suvival curves for patients with pancreatic head adenocarcinoma with and without delayed gastric emptying (DGE) after pancreaticoduodenectomy.

After multivariate analysis by binary logistic regression, RPD was the only independent factor associated with a lower incidence of DGE, as compared with OPD (Fig. 3). There was no survival difference for pancreatic head adenocarcinoma between the groups, with and without DGE after pancreaticoduodenectomy (Fig. 4).

## Discussion

Although not life-threatening, DGE is a common and exasperating complication. It usually resolves spontaneously with or without prokinetics, requiring several weeks or more with conservative management by NGT drainage^4^. The pathogenesis of DGE is multifactorial and poorly understood. It has been hypothesized that pyloric denervation, loss of pyloric pump, gastric dysrhythmia, antroduodenal ischemia, lack of motilin from duodenectomy, reduced activity of the motilin receptor, and inflammation, might lead to DGE. Patient-related factors, such as age, BMI, ASA class, male sex, smoking history, and intraoperative blood loss, have been shown to be associated with an increased incidence of DGE^1,5–9^.

There are several factors associated with DGE, such as PPPD, pancreaticogastrostomy, length of the preserved proximal portion of the duodenum, division of the right gastric artery, gastric/ duodenal devascularization, open approach, low volume of center experience, retrocolic route of gastroenteric reconstruction, high volume of preoperative gastric juice, long duration of gastric tube placement, no prokinetic agents, diabetic gastroparesis, history of cardiovascular or renal disease, periampullary cancers, preoperative biliary drainage, mechanical ventilation after operation, and intra-abdominal complications such as pancreatic leak, biliary leak, pancreatitis, and intra-abdominal abscess^1,2,4,7–9^. In this study, which involved a surgical approach with OPD, gastrointestinal upset (nausea/vomiting), malignancy, intraoperative blood loss > 200 c.c., and lymph node involvement were identified as risk factors for DGE after pancreaticoduodenectomy. However, none of these factors have been confirmed and universally accepted to be the contributing causes of DGE after pancreaticoduodenectomy. In our series, there were 70 cases of other benign lesion, and none of these benign diseases was associated with DGE. This low incidence of DGE in benign diseases could be a reflection of limited dissection of lymph node and scope of surgical resection or benign pathology itself.

The incidence of DGE in RPD is also variable, ranging from 4.5% to 56.1%^7,13,22,23^. This study showed a low rate (4.4%) of DGE in RPD with extracorporeal hand-sewn gastrojejunostomy via a small umbilical wound, which was created for extraction of the resected specimen. Thus, the stomach was pulled downward and positioned relatively vertical after gastrojejunal anastomosis in the patients with RPD. Jung et al.^7^ reviewed the videos of 192 RPDs with intra-corporeal gastrojejunal anastomosis and reported DGE in 41 (21.4%) patients (grade A, 15; grade B, 14; and grade C, 12). Technical variables contributing to decreased DGE on multivariate analysis included the type I gastrojejunal anastomosis flow angle (within 30° of vertical) between the stomach and efferent jejunal limb, greater length of the gastrojejunal anastomosis, and robotic-sewn, instead of stapler, anastomosis. The results have been supported by Sugiyama et al.^24^, Masui et al.^25^, and Murakami and Yasue^26^, who proposed that food would be easily facilitated downward by gravity through the gastrojejunal anastomosis in this straightened position, as the stomach might act as a passive conduit into the jejunum. These findings imply that a relatively “vertical and straight’’ gastrojejunostomy flow might contribute to the low incidence of DGE in our RPD patients, as shown in Fig. 2A.

After gastrojejunal anastomosis in our RPD patients, the final position of gastrojejunstomy was antecolic, antiperistaltic, and inframesocolic near the umbilicus region, as shown in Fig. 1. Miyazaki, et al.^4^ found that DGE rate was reduced when gastrojejunstomy was securely positioned at the inframesocolic point without angulation or torsion. Further, they suggested that, with respect to positioning, gastrojejunal anastomosis should be performed at the inframesocolic point, enabling separation of the gastrojejunostomy from the pancreaticojejunostomy. By separating these two anastomoses with the mesocolon, the influence of inflammation created after pancreaticoduodenal resection or related to the pancreatic fistula on the gastrojejunal anastomosis could be avoided or minimized. Therefore, they always take particular attention in positioning the stomach vertically and making the gastrojejunal anastomosis at the inframesocolic point on the left side of the abdominal cavity. We believe that antecolic, antiperistaltic, and inframesocolic gastrojejunostomy may keep the stomach away from the inflammatory area above the mesocolon and transverse colon, consequently reducing secondary DGE in our RPD patients.

In summary, there are three proposed mechanisms to lower the incidence of DGE in RPD patients. First, “food flow by gravity”: a relatively “vertical and straight” stomach in position after extracorporeal hand-sewn gastrojejunostomy via a small umbilical wound might facilitate food passage downward. Second, “separation of inflammation”: “inframesocolic, antecolic, and antiperistaltic (left-sided)” gastrojejunal anastomosis could keep the stomach away from the inflammatory area above the mesocolon and transverse colon. Third, “less inflammation/adhesion”: “smaller wound and less trauma”, less inflammation/adhesion.

The adverse effect of DGE on cancer-specific survival was claimed by Futagawa, et al.^27^. They assumed that a weakened immune system associated with poor nutrition might enhance the negative effects of DGE on overall survival. However, in this study, no impact of DGE on the survival outcomes of pancreatic head adenocarcinomas were observed after pancreaticoduodenectomy.

The present study is limited by the retrospective identification of the variable data. Another limitation is its non-randomized design. Moreover, selection bias could not be avoided, despite attempting to mitigate this by using multivariate logistic regression adjusted for the dissimilarities in baseline and treatment characteristics that occurred as a result of the non-randomized design.

In conclusion, RPD with extracorporeal hand-sewn antecolic, antiperistaltic, and inframesocolic gastrojejunostomy, involving careful downward positioning of the stomach, is associated with a low incidence of DGE. Jaundice, surgical approach with OPD, gastrointestinal upset (nausea/vomiting), malignancy, intraoperative blood loss > 200 c.c., and lymph node involvement are identified to be risk factors for DGE after pancreaticoduodenectomy. RPD under small-incision assisted gastrointestinal anastomosis is associated with a low incidence (4.4%) of DGE and is the only and most powerful independent predictor for DGE after multivariate analysis.

## Data Availability

The data that support the findings of this study are available on request from the corresponding author. The data are not publicly available due to privacy or ethical restrictions.

## Abbreviations

POPF: Postoperative pancreatic fistula
DGE: Delayed gastric emptying
OPD: Open pancreaticoduodenectomy
ISGPS: International Study Group of Pancreatic Surgery
PPPD: Pylorus-preservation pancreaticoduodenectomy
RPD: Robotic pancreaticoduodenectomy
IRB: Institutional Review Board
BMI: Body mass index
DM: Diabetes mellitus
ASA: American Society of Anesthesiologists
NGT: Nasogastric tube
POD: Postoperative day
PPH: Postpancreatectomy hemorrhage
